# Phosphorus, Nitrogen and Chlorophyll-*a* Are Significant Factors Controlling Ciliate Communities in Summer in the Northern Beibu Gulf, South China Sea

**DOI:** 10.1371/journal.pone.0101121

**Published:** 2014-07-02

**Authors:** Yibo Wang, Wenjing Zhang, Yuanshao Lin, Wenqing Cao, Lianming Zheng, Jun Yang

**Affiliations:** 1 Laboratory of Marine Biodiversity and Global Change (MBiGC), College of Ocean and Earth Sciences, Xiamen University, Xiamen, China; 2 Aquatic Ecohealth Group, Key Laboratory of Urban Environment and Health, Institute of Urban Environment, Chinese Academy of Sciences, Xiamen, China; University of Connecticut, United States of America

## Abstract

Ciliates (protozoa) are ubiquitous components of plankton community and play important roles in aquatic ecosystems in regards of their abundance, biomass, diversity and energy turnover. Based on the stratified samples collected from the northern Beibu Gulf in August 2011, species composition, abundance, biomass, diversity and spatial pattern of planktonic ciliates were studied. Furthermore the main environmental factors controlling ciliate communities were determined. A total of 101 species belonging to 44 genera and 7 orders (i.e., Oligotrichida, Haptorida, Euplotida, Sessilida, Pleurostomatida, Scuticociliatida and Tintinnida) were identified. The variation of ciliate communities was significant at horizontal level, but that was not at vertical level. Based on cluster analysis, ciliate communities were divided into three main groups. Redundancy analysis (RDA) revealed that Group A, existing in the waters with higher concentration of phosphorus and nitrogen, was dominated by *Tintinnidium primitivum*. Group B in the waters with lower temperature and chlorophyll-*a* concentration, was dominated by *Leegaardiella ovalis*. Group C, existing in the waters with higher temperature and chlorophyll-*a* concentration, was dominated by large *Strombidium* spp. and *Mesodinium rubrum*. Combining multiple analytic methods, our results strongly supported that phosphorus, nitrogen and chlorophyll-*a* were the most significant factors affecting the ciliate communities in the northern Beibu Gulf in summer. Concentration of phosphorus and nitrogen primarily influenced ciliate biomass, implying a potential impact of eutrophication on ciliate growth. The correlation with chlorophyll-*a* concentration, on one hand indicate the response of ciliates to the food availability, and on the other hand, the ciliates containing chloroplasts or endosymbionts may contribute greatly to the chlorophyll-*a*.

## Introduction

Ciliates are ubiquitous and significant components of microplankton communities in the ocean and play a crucial role in microbial food webs. They are important consumers of the pico- and nano- sized fractions of the plankton and the linkage in transferring energy from the microbial loop to higher trophic levels in the sea [1], [2]. Ciliates range in size from <10 to several hundred micrometres and may have higher metabolic rates and growth efficiencies compared with most metazoan organisms [3]. They have a substantial trophic impact in planktonic food webs because ciliates can graze 4% to 60% of net annual production, and they contribute significantly to heterotrophic biomass and production in the aquatic ecosystems. By virtue of their rapid growth rate and sensitive reaction to environmental changes, they have been considered as effective bioindicators of water quality and environmental contamination [2], [4], [5], [6].

However, there is still a shortage of data on marine ciliate ecology in tropical and subtropical seas up to now [7], [8]. Despite various investigations on coastal zone resources, ciliate protozoa as an important component were usually ignored for their small size, fast migration rate, fragile external membranes and the difficulty in laboratory culture. Chinese researches on planktonic ciliates were mostly conducted in the Yellow Sea [2], [4], [9], [10], [11] and South China Sea [7], [8], [12], [13], [14], [15]. In previous studies, ciliate samples for enumeration were mostly obtained by net method, which is unable to reveal the vertical distribution of ciliates and may cause an underestimate in ciliate population [3]. In this study, therefore, sampling bottles were used to obtain a more reliable estimate of species composition, abundance and biomass of planktonic ciliates.

The Beibu Gulf is a semi-enclosed gulf located in the northwest of the South China Sea surrounded by the land territories of China and Vietnam, with the port cities of Beihai, Qinzhou, Fangchenggang on the north coast, and Leizhou Peninsula on the east. As the fourth largest fish farming area in China, the Beibu Gulf has abundant marine resources due to the rich terrigenous nutrients carried by coastal rivers. Influenced by the East Asian Monsoon, southwesterly winds prevail in summer and northeasterly winds in winter [16], [17]. Controlled by multiple water masses and currents, the whole Beibu Gulf has complex hydrologic condition [17]. There have been a number of investigations on plankton community in the Beibu Gulf, however, data on ciliate communities are poor. The spatial pattern of ciliate communities has not yet been investigated in this area. We were particularly interested in the characteristics of ciliate communities formed in specific area. We wonder that of the many environmental factors, which are the most crucial to ciliate communities, although ciliate community structure is generally considered as a result of by various biotic and abiotic factors.

The aim of this study was (1) to characterize the species composition, abundance, biomass and diversity of the ciliate communities from the northern Beibu Gulf, (2) to reveal the distribution pattern of the ciliate communities at both horizontal and vertical levels, and (3) to determine the primary environmental factors that controlling the ciliate communities in the northern Beibu Gulf in summer.

## Materials and Methods

### Study stations and sampling

**Figure 1:**
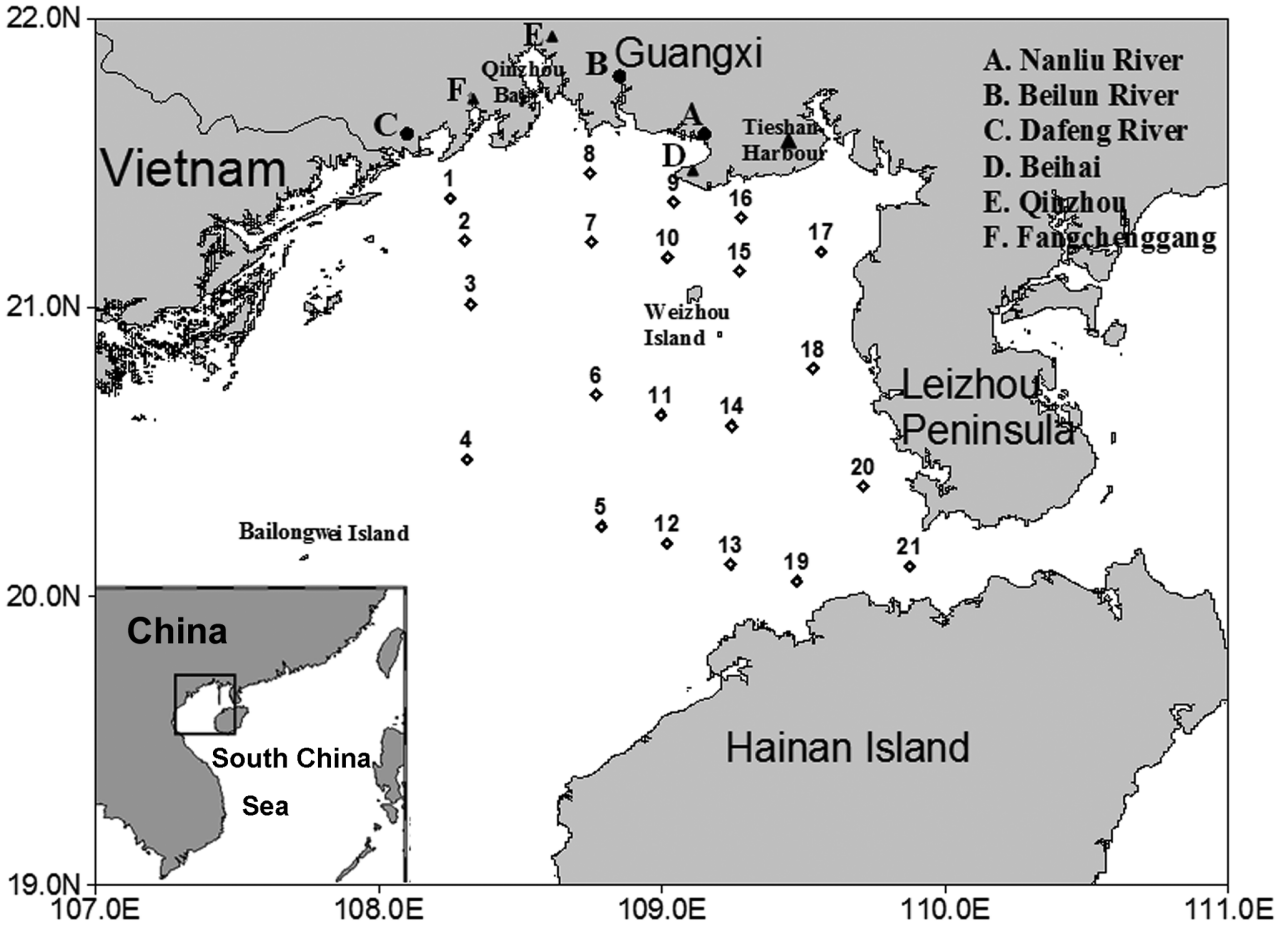
Sampling stations of planktonic ciliates in the northern Beibu Gulf in August 2011. (TIFF).

This study was carried out in the northern Beibu Gulf (108.25°∼109.88°E, 20.05°∼21.46°N), which was surrounded by Vietnam, Guangxi and Leizhou Peninsula of China (Figure 1). The cruise was carried out on the vessel “Tianying” from August 8th to 12th, 2011, and 21 stations were selected in the northern Beibu Gulf (Figure 1). Our sampling locations were not national park or other protected area of land and no specific permissions were required for our locations/activities. We confirm that the field studies did not involve endangered or protected species.

Multilayer water samples were collected from 21 stations using a Niskin bottle on the conductivity-temperature-depth (CTD) oceanic profilers (SBE-917). At the stations with water depths more than 30 m, water samples were collected at the surface, 10 m, 30 m, and the bottom, and with water depths less than 30 m, water samples were collected at the surface, 10 m and the bottom, while at the stations with water depths less than 10 m, water samples were taken at the surface and the bottom. A total of 62 water samples were obtained and fixed with acid Lugol's iodine solution (final concentration: 2%) in plastic bottles. A list of sampling layers in 21 stations is given in Table 1.

**Table 1:** Sampling layers in 21 stations for planktonic ciliates in the northern Beibu Gulf.

Station	Sampling layers
1	surface, 10 m, bottom
2	surface, 10 m, bottom
3	surface, 10 m, bottom
4	surface, 10 m, 30 m, bottom
5	surface, 10 m, 30 m, bottom
6	surface, 10 m, 30 m, bottom
7	surface, 10 m, 30 m
8	surface, bottom
9	surface, bottom
10	surface, 10 m, bottom
11	surface, 10 m, bottom
12	surface, 10 m, bottom
13	surface, 10 m, bottom
14	surface, 10 m, bottom
15	surface, 10 m, bottom
16	surface, bottom
17	surface, bottom
18	surface, bottom
19	surface, 10 m, bottom
20	surface, 10 m, bottom
21	surface, 10 m, 30 m, bottom

Sampling depth, water temperature and salinity were measured in situ using an onboard CTD (SBE-917) via probes. Chlorophyll-*a* concentration was measured using a Turner Fluorometer (10-AU-005). PH, dissolved oxygen (DO), total organic carbon (TOC), nitrate nitrogen (NO_3_-N), nitrite nitrogen (NO_2_-N), ammonium nitrogen (NH_4_-N), total nitrogen (TN), active phosphorus (AP), total phosphorus (TP) and active silicon (ASi) were determined according to the standard methods established in the Offshore Marine Chemical Survey Technical Regulations [18].

### Identification and enumeration

For identification and enumeration, 1 L of Lugol's fixed seawater was settled for 48 hours resulting in 15 mL of concentrated sample [19]. 0.1 mL of well-mixed concentrated sample was taken and identified in a microscope slide. A total volume of 1 mL concentrated sample were counted and identified under a light microscope at a magnification 400×. Tintinnids were identified by lorica morphology and species description according to Hada [20], [21], [22], Kofoid and Campbell [23], [24], Lynn [25] and Nie [26]. Aloricate ciliates were identified following Leegaard [27], Lynn [25] and Song et al [28]. All ciliates were finally identified to the lowest possible taxa. The individuals which could not be identified with Lugol's-fixed samples were picked out and identified using protargol impregnation after re-fixating with Bouin's solution [29]. The classification system was mainly referred to Lynn [25].

Body volumes of ciliate cells were determined from measuring of their body size and applying volume equations of approximate geometric shape. Conversion factor of C: Vol was 0.19 pg C·µm^-3^ for cells preserved with 2% Lugol's iodine [30]. For tintinnids, the cell volumes were approximately equal to 1/3 of the loricate volumes [8], thus conversion factor of C: Vol was 0.063 pg C·µm^−3^ for loricate cells.

### Data analyses

Species number, abundance and biomass were calculated for each sample. The horizontal and vertical distribution patterns of planktonic ciliates were displayed using software surfer 8 and ocean data view (ODV).

Shannon-Weiner diversity index (*H*′) for each sample and dominance (*Y*) for ciliate species were calculated by the following equations:
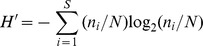



*Y* = (*n_i_*/*N*)·*f_i_,* Where *n_i_*  =  abundance of the *i*th species; *S*  =  total number of species; *N*  =  total number of individuals; *f_i_*  =  occurrence of the *i*th species.

Depth-averaged abundance and biomass were calculated by the integration formula:
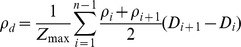



Where *ρ_d_*  =  depth-averaged abundance/biomass; Z_max_  =  maximum depth of the station; *n*  =  maximum number of layers; 1≤*i*≤*n*-1; *ρ_i_*  =  density in the *i*th water layer; D*_i_*  =  depth of the *i*th water layer.

Cluster analysis was used to investigate ciliate community structure using the PRIMER v5.0 (Plymouth Routines In Multivariate Ecological Research) package according to species composition and abundance [31]. To ensure reliability, samples with observed individuals less than 100 were excluded from the biotic data before clustering. Bray-Curtis similarity matrix was computed on square root-transformed biotic data and Euclidean distance matrix was on ln(x+1)-transformed abiotic data. Group-average clustering was used to separate the ciliate communities into several assemblages, which represented natural groupings of samples (Group A, B, C, etc.), each comprising samples with similar species and abundances. Grouping at horizontal level and vertical level were also conducted. Groups at horizontal level (Group 1, 2, 3, etc.) were classified by clustering based on species composition and their depth-averaged abundances in 21 stations, and groups at vertical level (Group I, II, III, etc.) were classified based on water depth (Group I: 0<water depth≤0.5 m, Group II: 0.5<water depth≤10 m, Group III: 10<water depth≤30 m, Group IV: water depth>30). Variances between groups at different levels were tested using the submodule ANOSIM (analysis of similarities) in PRIMER v5 (global *R* values range from 0 to 1, and higher global *R* values are more significant.). Species with a cumulative contribution of 90% to the average Bray-Curtis similarity within each group was analyzed using the submodule SIMPER (Similarity Percentage Analysis). Additionally, the significance of biota-environment correlation was checked by using the RELATE. A potential relationship between biotic data and environmental data was explored using the BIOENV (biota-environment). To obtain a more detailed relationship between ciliate communities and environmental factors, redundancy analysis (RDA) was conducted by using Canoco for Windows 4.5 package [32]. Under forward selection, significance of correlation with environmental variables was determined by Monte Carlo permutation test (999 permutations). RDA ordination plots were generated based on square root-transformed species-abundance data and ln(x+1)-transformed environmental data. Spearman correlation analysis between biotic data and environmental variables was conducted by using statistical program SPSS v17.0.

## Results

### Species composition

A total of 101 species belonging to 44 genera and 7 orders (Oligotrichida, Haptorida, Euplotida, Sessilida, Pleurostomatida, Scuticociliatida and Tintinnida) were identified. A list of all observed species from 7 orders is given in Table S1. The most diverse orders were Tintinnida (43 species) and Oligotrichida (40 species), and the most abundant orders were Oligotrichida (2,542.44±248.24 ind·L^−1^) and Haptorida (976.33±120.82 ind·L^−1^). As regards ciliate biomass, Tintinnida (2.46±0.65 µg C·L^−1^) and Oligotrichida (2.31±0.25 µg C·L^−1^) were the most contributive orders. The numbers of loricate and aloricate species in all samples were 43 and 58 respectively. Their average abundances were 571.81±133.61 ind·L^−1^ and 3,634.96±461.64 ind·L^−1^, respectively, and average biomasses were 2.46±0.65 µg C·L^−1^ and 2.73±0.29 µg C·L^−1^, respectively.

The ciliate communities were dominated by 8 species (dominance *Y*≥0.02), i.e. *Mesodinium rubrum* (*Y* = 0.1562), *Strombidium inclinatum* (*Y* = 0.1018), *Mesodinium velox* (*Y* = 0.0502), *Strombidium parastylifer* (*Y* = 0.0481), *Strombidium conicum* (*Y* = 0.0411), *Tintinnidium primitivum* (*Y* = 0.0405), *Leegaardiella ovalis* (*Y* = 0.0255), *Strombidium emergens* (*Y* = 0.0229). They occurred in majority of the stations, and the cumulative abundance of them exceeded 60% of the total in August. Among these dominant species, *Mesodinium rubrum* occurred most frequently that they were observed in 93.55% of the samples; *Tintinnidium primitivum* was the only loricate species and had a widespread distribution as well.

### Horizontal distribution

#### Horizontal distribution of ciliate abundance

**Figure 2:**
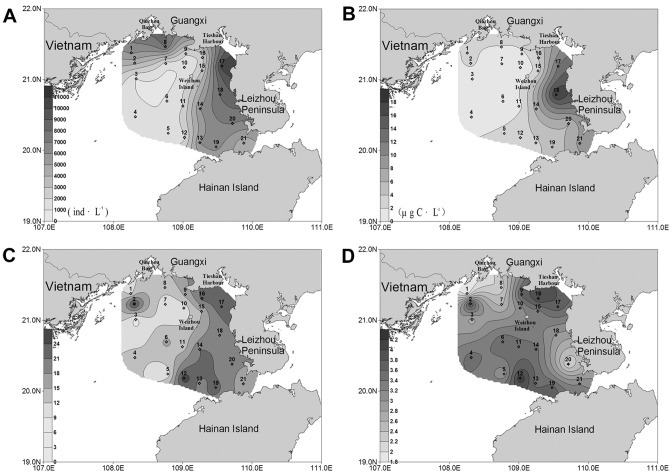
Horizontal distribution of planktonic ciliates in the surface layer of the northern Beibu Gulf. (A) Horizontal distribution of abundance. (B) Horizontal distribution of biomass. (C) Horizontal distribution of species number. (D) Horizontal distribution of diversity. The horizontal axis and the vertical axis expressed the gradients in longitude and latitude, respectively. (TIFF).

**Figure 3:**
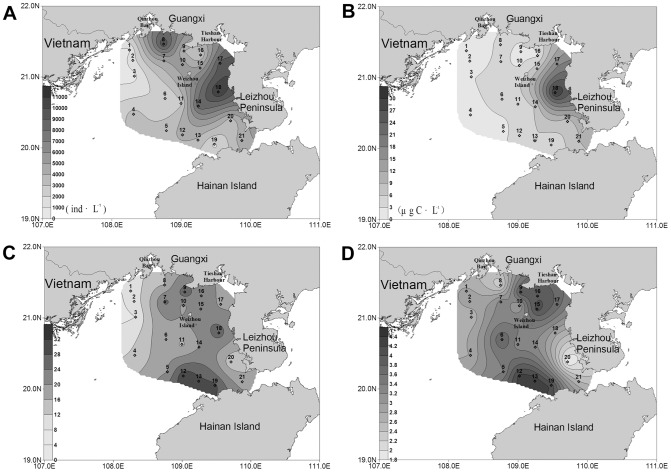
Horizontal distribution of planktonic ciliates in the bottom layer of the northern Beibu Gulf. (A) Horizontal distribution of abundance. (B) Horizontal distribution of biomass. (C) Horizontal distribution of species number. (D) Horizontal distribution of diversity. The horizontal axis and the vertical axis expressed the gradients in longitude and latitude, respectively. (TIFF).

**Figure 4:**
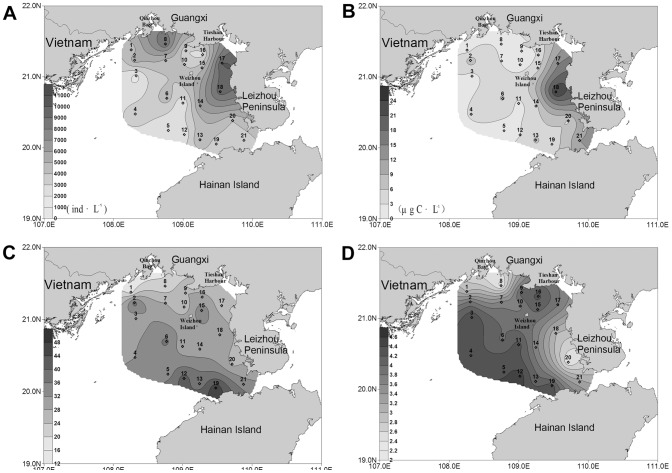
Horizontal distribution of planktonic ciliates in the water columns of the northern Beibu Gulf. (A) Horizontal distribution of abundance. (B) Horizontal distribution of biomass. (C) Horizontal distribution of species number. (D) Horizontal distribution of diversity. The horizontal axis and the vertical axis expressed the gradients in longitude and latitude, respectively. (TIFF).

In August, the ciliate abundance in the surface water in the nearshore area was higher than that in the offshore (Figure 2A). The two concentrated zones were the coastal region of Guangxi and the west coastal region of Leizhou Peninsula, especially the area adjacent to Tieshan Harbor. The highest abundance (10.64×10^3^ ind·L^−1^) was observed in the bottom at station 17. With the increasing distance from the coast, the abundance of ciliates gradually decreased. The lowest value (0.99×10^3^ ind·L^−1^) was observed at station 3. The distribution in the bottom layer showed a similar trend with surface water (Figure 3A). The highest abundance (10.95×10^3^ ind·L^−1^) occurred in the bottom at station 18, while the lowest value (0.74×10^3^ ind·L^−1^) occurred at station 3. Concerning the depth-averaged abundance, the high values was observed around stations 17 and 18, while the lowest value was recorded at station 3 (Figure 4A).

#### Horizontal distribution of ciliate biomass

The depth-averaged biomass decreased westwards from the west coast of Leizhou Peninsula (Figure 4B). The same was true for the biomass in each layer. In the surface water, the highest biomass (18.26 µg C·L^−1^) was observed at station 18 (Figure 2B), while the lowest value (0.48 µg C·L^−1^) occurred at station 3. In the bottom water, the ciliate biomass peaked at station 18 (30.53 µg C·L^−1^), and the lowest value (0.45 µg C·L^−1^) occurred at station 1 (Figure 3B).

#### Horizontal distribution of species number

The species numbers of ciliates were variable among stations. In the surface water, a conspicuous westwards decrease of the species numbers was showed, ranging from 31 (station 12) in the east to 8 (station 3) in the west (Figure 2C). The bottom layer showed a similar trend that the species numbers in the west area were less than in the east (Figure 3C). For the species number in each station, however, higher values were concentrated in the area north of Hainan Island while lower values were recorded in the coastal waters of Guangxi-Vietnam (Figure 4C). There were only 18 species identified at station 1, while as many as 47 species were observed at station 19.

#### Horizontal distribution of diversity

In contrast with the distribution trend of ciliate abundance (Figure 2A, 3A, 4A), Shannon-Weiner diversity of each station was obviously lower in the nearshore waters than in the offshore waters (Figure 4D), and the same was true for the diversity in each layer (Figure 2D and 3D). The zones with lower values displayed patchy distributions. One patch was around station 8 at the mouth of Qinzhou Bay in Guangxi, and the other was around station 20 in the west coast of Leizhou Peninsula. The area nearby Tieshan Harbor as well as the large area southwest of Weizhou Island displayed higher values. In the surface water, Shannon-Weiner diversity minimized (1.82) at station 1 and maximized (4.17) at station 12 (Figure 2D). In the bottom layer, the minimum (1.81) was observed at station 20, while the maximum (4.35) was at station 13 (Figure 3D).

### Vertical distribution

In this study, four vertical sections were picked out to investigate the vertical distribution trend of ciliate abundance, biomass, species number and Shannon-Weiner diversity with increased water depth and distance from the coast. Sections 1–4 (stations 1, 2, 3, 4) and 5–8 (stations 5, 6, 7, 8) were in longitude direction, while sections 4–19 (stations 4, 5, 12, 13, 19) and 3–20 (stations 3, 6, 11, 14, 20) were in latitude direction.

**Figure 5:**
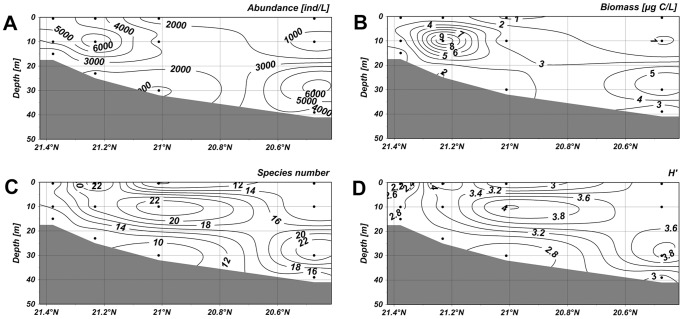
Vertical distribution of planktonic ciliates in the section 1–4. (A) Vertical distribution of abundance. (B) Vertical distribution of biomass. (C) Vertical distribution of species number. (D) Vertical distribution of diversity. The horizontal axis and vertical axis expressed the gradients in latitude and water depth, respectively. (TIFF).

**Figure 6:**
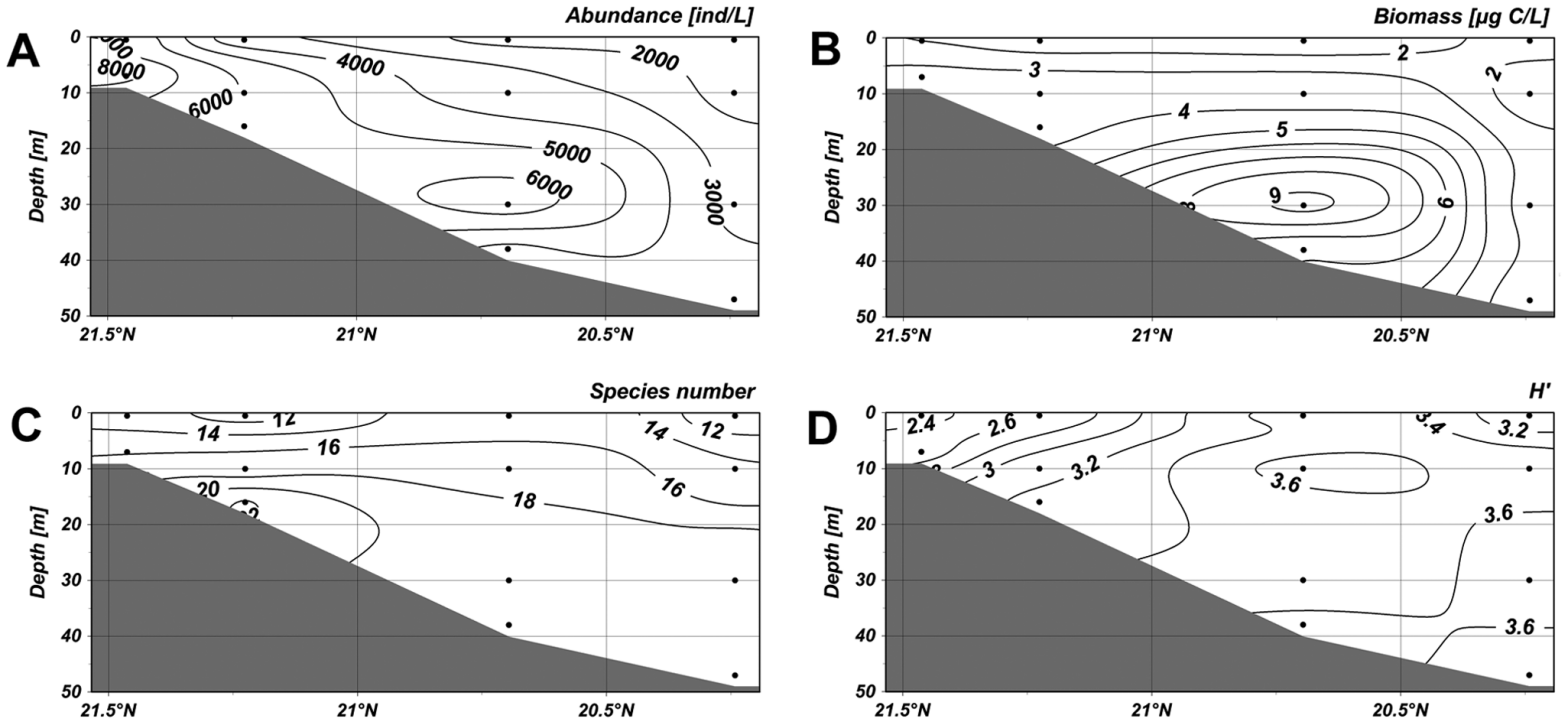
Vertical distribution of planktonic ciliates in the section 5–8. (A) Vertical distribution of abundance. (B) Vertical distribution of biomass. (C) Vertical distribution of species number. (D) Vertical distribution of diversity. The horizontal axis and vertical axis expressed the gradients in latitude and water depth, respectively. (TIFF).

**Figure 7:**
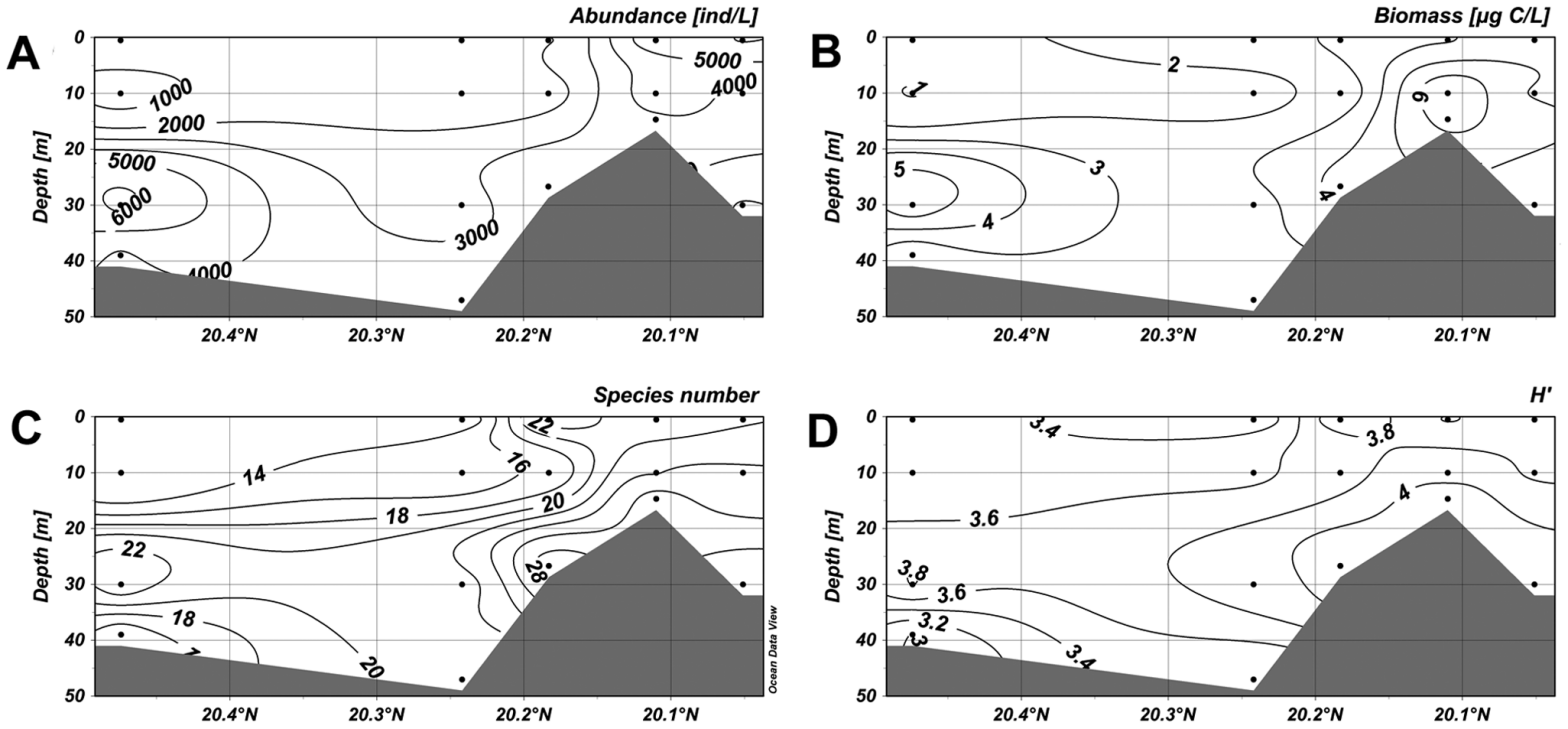
Vertical distribution of planktonic ciliates in the section 4–19. (A) Vertical distribution of abundance. (B) Vertical distribution of biomass. (C) Vertical distribution of species number. (D) Vertical distribution of diversity. The horizontal axis and vertical axis expressed the gradients in latitude and water depth, respectively. (TIFF).

Along the section 1–4, abundance of ciliates were low in the surface water but it maximized (8.53×10^3^ ind·L^−1^) at a depth of 10 m in the nearshore waters (Figure 5A). The second highest value was observed in the layer of 30 m in the offshore waters. The distribution of the biomass, in accord with abundance, showed high values at a depth of 10 m in the nearshore waters and at about 30 m offshore (Figure 5B). In the section 5–8, the abundance peaked with a higher value 9.92×10^3^ ind·L^−1^ at a depth of 10 m (Figure 6A). The high values of biomass, however, only occurred at a depth of 30 m in the offshore waters (Figure 6B). The abundance of ciliates in the section 4–19 showed a maximum value (6.83×10^3^ ind·L^−1^) at a depth of 30 m in offshore waters, and higher values were also observed at the surface layer in the nearshore waters (Figure 7A). The biomass maximized at a depth of 10 m in the nearshore waters and the second highest biomass occurred at a depth of 30 m in the offshore waters (Figure 7B). In the section 3–20, the highest abundance and biomass were both occurred at a depth of about 30 m in offshore waters.

**Figure 8:**
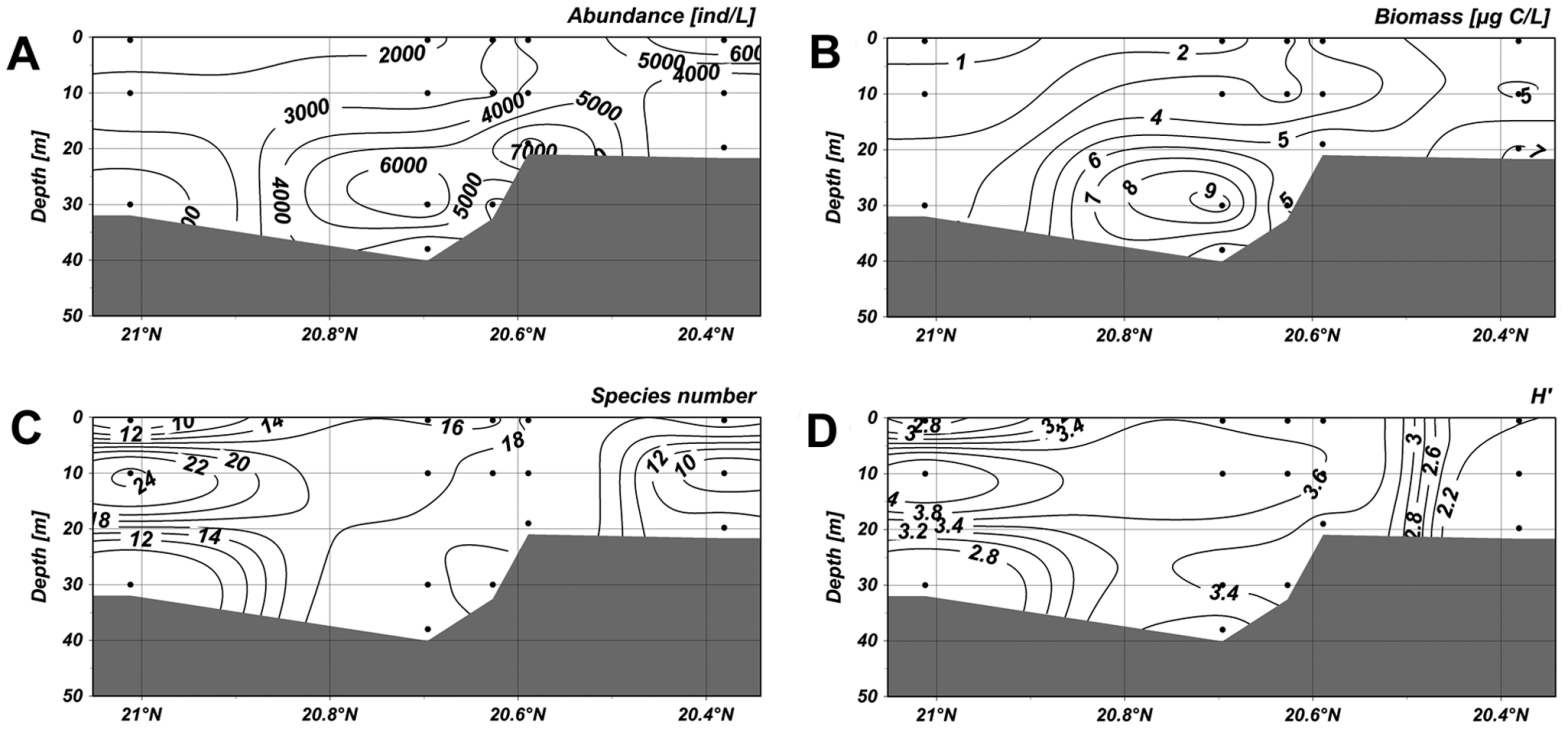
Vertical distribution of planktonic ciliates in the section 3–20. (A) Vertical distribution of abundance. (B) Vertical distribution of biomass. (C) Vertical distribution of species number. (D) Vertical distribution of diversity. The horizontal axis and vertical axis expressed the gradients in latitude and water depth, respectively. (TIFF).

High species numbers in the section 1–4 gathered at depths of 10 m and 30 m in the offshore area (Figure 5C). In the section 5–8, high species numbers appeared below the depth of 10 m in the offshore waters (Figure 6C). In the section 4–19, the higher values were mostly observed in the nearshore waters, and the values were also relatively high at a depth of more than 20 m in the offshore waters (Figure 7C). In the section 3–20, lower species numbers were recorded in the nearshore waters while higher values were observed at about 10 m in the offshore waters (Figure 8C). Areas with higher Shannon-Weiner diversity indices were almost those with higher species numbers (Figure 5–8).

A conspicuous consistent trend could be found out between the vertical distributions of ciliate abundance and biomass. The aggregated areas were displayed at a depth of 10 m in the nearshore waters, while descended to about 30 m in the offshore waters. No consistent trend was found between species number and abundance/biomass, however, the vertical distributions of species number and diversity were almost the same.

### Spatial pattern of ciliate communities

The variations of horizontal distribution and vertical distribution were studied. At the horizontal level, cluster analysis on the data of species composition and their depth-averaged abundance divided 21 stations into four groups at a similarity level of 50.11%: Group 1 comprised 3 stations (18, 20, 21) located in the west coast of Leizhou Peninsula; Group 2 included only one station (17) situated nearby Tieshan Harbour; Group 3 covered most off-shore stations (2–7, 9–16, 19) in the southwest of our study area; Group 4 included 2 inshore stations (1, 8) by the coast of Guangxi. Based on the groupings above, all 62 samples were classified into four groups. A relatively significant difference between groups was tested at horizontal level (global *R* = 0.376, *P*<0.001). At the vertical level, all samples were also divided into four groups in terms of the depth of each sample. Group I consisted of 21 samples from a depth not more than 0.5 m; Group II comprised 20 samples from a depth more than 0.5 m but not more than 10 m; Group III comprised 17 samples from a depth more than 10 m but not more than 30 m, and Group IV included the other 4 samples which were collected from a depth more than 30 m. The difference between groups was not so significant (global *R* = 0.095, *P* = 0.002), suggesting a less conspicuous variation of the vertical distribution than of the horizontal distribution.

**Figure 9:**
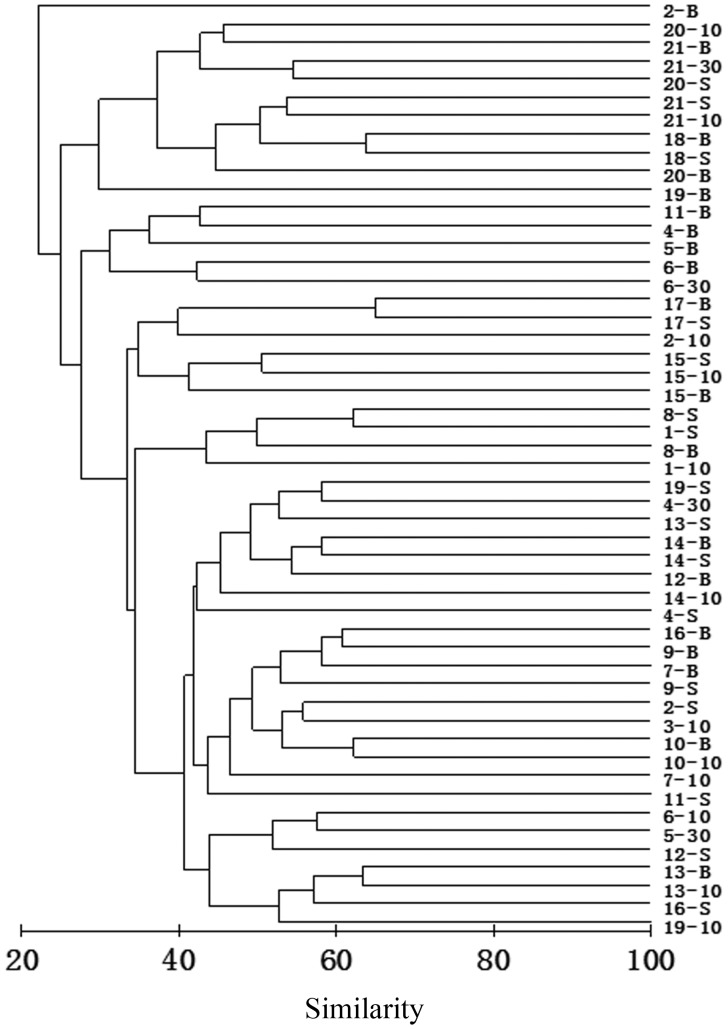
Result of cluster analysis. The result was based on Bray-Curtis similarities from square root-transformed biotic data of 51 effective samples in the northern Beibu Gulf in August 2011. The numbers indicated the sampling stations. -S  =  surface-layer sample; −10 = 10 m-layer sample; −30 = 30 m-layer sample; -B  =  bottom-layer sample. (TIFF).

Cluster analysis separated the ciliate samples in which individuals were not less than 100 into several groups, each comprising samples with similar species composition and biomass. A dendrogram showed that 51 effective samples were divided into four clusters at a similarity level of 27.51% (Figure 9). Thus three main groups were obtained excluding the bottom sample of station 2. Group A was comprised of samples of stations 18–21 west of Leizhou Peninsula and at the mouth of Qiongzhou Strait; Group B consisted of off-shore stations 4, 5, 6, 11 in the southwest area; Group C consisted of the most samples from the southwest off-shore stations. Group C was further divided into three subgroups. Subgroup a consisted of samples of stations 15, 17 nearby Tieshan Harbour and of station 2. Subgroup b consisted of samples of stations 1 and 8. Subgroup c consisted of the other samples from the southwest area.

Species that were most contributive to each group were determined using SIMPER analysis. In Group A, *Tintinnidium primitivum*, the only tintinnid of the dominant species, was with a greatest contribution of 44.28%, followed by *Tintinnopsis schotti* (19.34%) and *Laboea strobili* (13.78%). Major contributors to Group B were *Leegaardiella ovalis* (20.72%), *Tintinnidium primitivum* (17.00%) and *Strombidium paracalkinsi* (16.81%). Group C, comprising of most samples, was the largest group, and the most contributive species to Group C were *Omegastrombidium jankowskii* (14.00%), *Strombidium conicum* (11.67%), *Mesodinium rubrum* (10.82%). Within Group C, *Omegastrombidium jankowskii* (32.34%) and *Strombidium conicum* (10.57%) were most contributive to Subgroup a, *Strombidium inclinatum* (51.71%) and *Strombidium conicum* (15.93%) were most contributive to Subgroup b, whilst *Omegastrombidium jankowskii* (12.78%) and *Mesodinium rubrum* (10.73%) were more important in Subgroup c. The bottom sample from station 2 was not included in any of these groups, mainly because of the dominance of *Leprotintinnus simplex*, *Stenosemella parvicollis* and *Strombidium conicum*.

### Correlations between ciliate communities and environmental factors

RELATE analysis revealed a significant correlation between the spatial variation of ciliate communities and the variation of environmental data (*R* = 0.109; *P* = 0.004). BIOENV analysis suggested the combination of environmental variables that were most correlative with the spatial variation of ciliate communities were: AP, TP, NO_2_-N, NO_3_-N and NH_4_-N (Table 2). Among them TP was the only variable included in all the top 10 matches, indicating TP was the key environmental variable that significantly influenced the variation of ciliate communities. Spearman correlation analysis indicated that the ciliate abundance, biomass and species numbers were significantly and positively correlated with concentration of chlorophyll-*a*, and ciliate biomass also exhibited a significantly positive correlation with concentrations of phosphorus and nitrogen nutrients (AP, TP, NO_2_-N) (Table 3).

**Table 2:** Result of biota-environment (BIOENV) analysis.

Rank	*R*	Environmental variables	*P*
1	0.435	AP, TP, NO_2_-N	0.01
2	0.427	TP, NO_2_-N	0.01
3	0.407	AP, TP, NO_2_-N, NH_4_-N	0.01
4	0.404	AP, TP, NO_2_-N, NO_3_-N	0.01
5	0.400	TP, NO_2_-N, NH_4_-N	0.01
6	0.399	TP, NO_2_-N, NO_3_-N	0.01
7	0.391	AP, TP, NO_2_-N, NO_3_-N, NH_4_-N	0.01
8	0.387	TP, NO_2_-N, NO_3_-N, NH_4_-N	0.01
9	0.383	AP, TP	0.01
10	0.380	AP, TP, NO_3_-N	0.01

The result shows the top 10 matches of environmental variables with spatial variance of ciliate community.

**Table 3:** Spearman correlations between biotic data and environmental variables in the northern Beibu Gulf.

Biotic Data	D	T	Sal	DO	pH	TOC	ASi	AP	TP	NO_2_-N	NO_3_-N	NH_4_-N	TN	TChl *a*	Pico-	Nano-	Micro-
**Community parameters**																	
Abundance	−0.019	0.303*	−0.271*	−0.148	−0.054	0.012	−0.101	0.137	0.029	0.207	0.009	0.226	−0.084	0.602**	0.128	0.538**	0.402**
Biomass	0.176	−0.072	−0.022	−0.172	−0.120	0.151	−0.237	0.392**	0.378**	0.342**	0.181	0.065	−0.041	0.653**	−0.024	0.650**	0.365**
Species number	0.111	0.043	0.022	0.051	0.301*	0.159	−0.170	0.099	−0.119	0.020	−0.173	0.211	−0.010	0.429**	0.139	0.363**	0.250*
*H′*	0.001	−0.070	0.179	0.247	0.485**	0.163	−0.156	−0.167	−0.301*	−0.293*	−0.410**	0.030	−0.051	0.099	0.295*	0.065	−0.069
**Dominant species**																	
*Leegaardiella ovalis*	0.465**	−0.397**	0.558**	−0.184	−0.015	−0.224	0.163	0.281*	−0.197	0.126	−0.016	−0.031	−0.198	0.089	0.235	0.141	−0.242
*Strombidium emergens*	−0.135	0.229	−0.127	0.278*	0.159	0.269*	−0.157	−0.185	−0.269*	−0.303*	−0.196	0.194	0.034	0.279*	0.221	0.168	0.270*
*Strombidium inclinatum*	−0.126	0.307*	−0.302*	−0.040	0.111	−0.044	0.024	−0.134	−0.238	−0.021	−0.255*	0.098	−0.051	0.243	0.154	0.206	0.062
*Tintinnidium primitivum*	0.430**	−0.402**	0.215	−0.303*	−0.272*	−0.058	−0.074	0.621**	0.494**	0.507**	0.496**	−0.087	−0.106	0.288*	−0.221	0.391**	0.074
*Mesodinium rubrum*	−0.064	0.311*	−0.281*	−0.147	−0.120	0.180	−0.234	0.168	0.143	0.229	−0.017	0.073	−0.021	0.585**	−0.065	0.520**	0.495**
*Strombidium parastylifer*	0.027	0.235	−0.231	−0.262*	−0.046	−0.079	0.041	0.332**	0.125	0.337**	0.045	0.245	−0.094	0.517**	0.013	0.520**	0.231
*Mesodinium velox*	−0.042	0.284*	−0.264*	−0.243	0.002	−0.173	0.080	0.182	0.023	0.271*	−0.025	0.244	−0.028	0.293*	0.014	0.301*	0.260*
*Strombidium conicum*	−0.245	0.192	−0.124	−0.030	0.099	0.026	−0.065	−0.129	−0.070	0.046	−0.173	0.228	0.012	0.209	0.262*	0.173	0.090

*P<0.05; **P<0.01. TChl *a*-total chlorophyll-*a*; Pico-picochlorophyll-*a*; Nano-nanochlorophyll-*a*; Micro-microchlorophyll-*a*.

**Figure 10:**
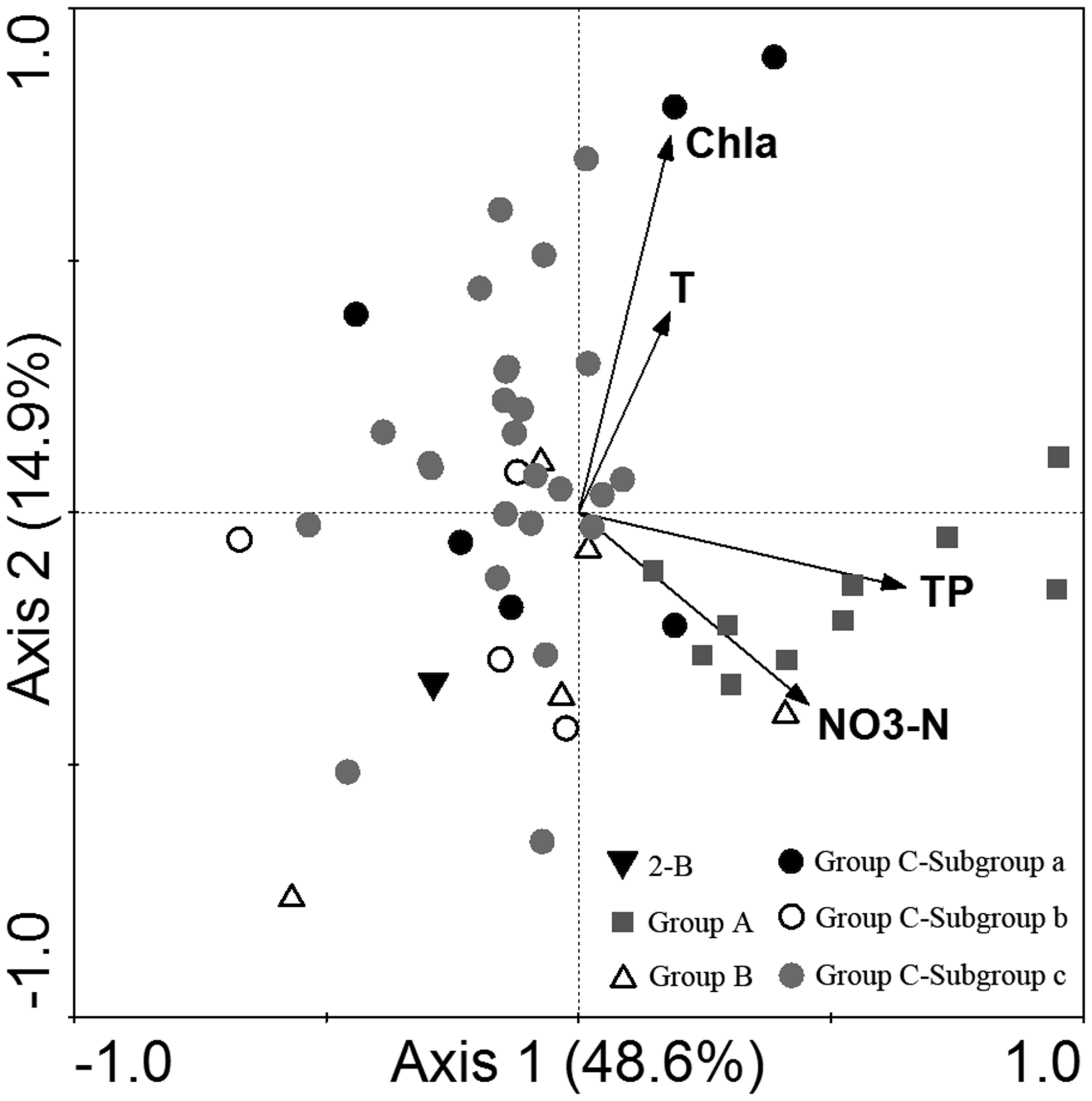
Redundancy analysis (RDA) ordination plot. The ordination plot shows the relationship between ciliate communities and significant environmental variables. The result was based on square root-transformed biotic and log-transformed abiotic data of 51 effective samples in the northern Beibu Gulf in August 2011. Axis 1 and Axis 2 explained 48.6% and 14.9% of the species-environment relationship respectively. T-water temperature; Chl *a*- chlorophyll-*a*; Sal-salinity; 2-B  =  bottom sample of station 2. (TIFF).

A species-abundance matrix in detrended correspondence analysis (DCA) resulted in the biggest gradient length 3.320. Redundancy analysis (RDA) method was selected for the analysis of sample-environment relationship. Among 14 environmental factors for study, four most significant factors, i.e. TP, NO_3_-N, chlorophyll-*a* and water temperature, were picked out by forward selection. All the four canonical axes jointly explained 77.7% (significant factors) of the species-environment relationship. The first and second axes which jointly explained 63.5% of the relationship were exhibited on the RDA two-dimensional plot (Figure 10). Group A was at a considerably high level of TP and NO_3_-N. Group B showed lower levels of water temperature and chlorophyll-*a*. By contrast, Group C displayed a higher level of water temperature and chlorophyll-*a*. In Group C, there was difference between subgroups. Water temperature and chlorophyll-*a* concentration in Subgroup a and c were relatively high, whilst those in subgroup b were lower.

## Discussion

### Spatial pattern of ciliate communities

A high value of diversity index suggests a more healthy ecosystem, while a low value suggests a less healthy or degraded ecosystem [33]. Shannon-Weiner diversity index *H′* is one of the most widely used diversity indices for measuring biodiversity. The Shannon-Weiner diversity index of ciliate communities in the northern Beibu Gulf remarkably varied. It was much lower in coastal waters than in offshore waters, implying a great impact of human activity to the water quality and the health of plankton ecosystem.

In addition, ANOSIM analysis revealed that the variation of ciliate communities was significant at horizontal level, but it was not significant at vertical level. The strong tide in the Gulf gives the basic force for vertical and horizontal mixing, making the waters mix quickly [16]. At vertical level, the water is relatively shallow that the maximum depth is less than 100 m. Seawater is well mixed in the vertical direction so that the physical and chemical conditions in the whole water columns vary slightly. At horizontal level, in contrast, the northern Beibu Gulf covers a vast expanse and is affected by different water masses and currents. The physical and chemical characters of seawater and how badly it is affected by human activities differ greatly. As a result, the ciliate communities in the study area exhibited a relatively homogeneous distribution vertically but varied greatly among stations.

### Relationship between ciliate communities and environmental factors

*Tintinnidium primitivum* is a typical species living in the cold water mass under the thermocline. In our survey, *T*. *primitivum* did existed in the cold water mass in the southwest area, verifying its positive correlation with water depth and negative correlation with temperature (Table 3). However, a much greater abundance of *T*. *primitivum* was recorded in the coastal waters west of Leizhou Peninsula. *T*. *primitivum* was also proved to be positively correlated with nitrogen and phosphorus (AP, TP, NO_2_-N, NO_3_-N) (Table 3), revealing its preference for nitrogen and phosphorus.

Among the dominant species, two (*Mesodinium rubrum* and *Mesodinium velox*) were in genus *Mesodinium* and they contributed significantly to the total abundance. *Mesodinium* spp. can form a high density in a short time in eutrophic waters and cause *Mesodinium* spp. bloom [7]. *M*. *rubrum* is an autotrophic species containing a permanent symbiont consisting of many chloroplasts [34]–[36]. It can utilize light and nutrients to carry out photosynthesis and has strong ability of motility and vertical migration [35]. The highest abundance (4.46×10^3^ ind·L^−1^) of *M*. *rubrum* was observed in the surface waters west of Leizhou Peninsula, where a high concentration of chlorophyll-*a* was measured. Our results showed that *M*. *rubrum* was significantly correlated with total chlorophyll-*a* as well as nano- and micro- sized chlorophyll-*a* concentration. The contribution of *M. rubrum* to the chlorophyll-*a* concentration may be important. And this may also suggest a preference of *M. rubrum* to eutrophic conditions.

The abundance, biomass and species number of ciliate communities were found significantly correlated to chlorophyll-*a* concentration, especially the nano- and micro- chlorophyll-*a*. For one thing, heterotrophic planktonic ciliates respond rapidly to their food availability in the aquatic environment. For another, autotrophic ciliates (e.g. *Mesodinium rubrum*) and mixotrophic ciliates (e.g. *Laboea strobila*, *Strombidium stylifer*) contain chloroplasts or symbiont consisting of chloroplasts [34]–[38]. They may contribute greatly to the chlorophyll-*a* in the northern Beibu Gulf, as in many coastal and estuarine ecosystems [39]–[41].

Besides, the spatial variation of ciliate communities was significantly correlated with the variables of phosphorus and nitrogen nutrients (i.e. TP, AP, NO_2_-N and NO_3_-N), suggesting a potential impact of eutrophication in estuarine and coastal waters on the ciliate communities. Nutrients directly affect the photosynthesis of photosynthetic ciliates, while heterotrophic ciliates are impacted by nutrients indirectly, through the effect on phytoplankton growth. Spearman analysis also indicated high concentrations of phosphorus and nitrogen nutrients primarily influenced ciliate biomass. It means the eutrophic state is more likely to cause an increase in ciliate size rather than their quantity or diversity.

The ciliate community is affected by multiple environmental factors. Both top-down and bottom-up interactions may play a role in population control of ciliates [42]. The response of ciliate community to nutrients and chlorophyll-*a* concentration also provided an evidence for ciliates as bioindicators.

### Relationship between ciliate communities and water masses

It should be noted that planktonic ciliates are creatures that lack of ability of independent movement and drift with the water currents. Their spatial distribution largely depends on the water currents and masses in study area. The whole Beibu Gulf can be generally divided into several masses, i.e. the coastal water, the outer Beibu Gulf water, the mixed water, the bottom water and so on [16], [17]. The coastal water mass with low salinity mainly affects the northwest of the bay, and the South China Sea water mass with high salinity covers the south of the bay. Meanwhile, the northern Beibu Gulf is also influenced by coastal currents and Qiongzhou Strait West Current.

In the Beibu Gulf, the southwesterly wind strengthens the northward flow west of Hainan Island in summer, causing the strongest intrusion of the South China Sea surface water from the mouth of the gulf, which is characterized by high salinity [17]. Affected by the surface and subsurface water, the bottom water which originates from the coastal water is limited below the thermocline and covers only a small area east of Bailongwei Island [16], [43]. In our study, the thermocline was observed at a depth of 10–30 m in stations 4, 5, 6 and 11. Group B may represent an offshore community which was mainly controlled by the bottom cold water. *Leegaardiella ovalis*, a small sized species, which prefers high salinity and low temperature, prevailed in this group.

The diluted water, characterized by low salinity and relatively high temperature and dissolved oxygen, is mainly formed by the river discharges from the Nanliu River, the Beilun River, and the Dafeng River in Guangxi. Interacted with the high-salinity South China Sea water, the diluted water is restricted to a small area off the Guangxi-Vietnam coast. Most area north of 20°N and west of Leizhou Peninsula was occupied by the mixed water [16]. The mixed water is a mixture between the diluted water and the surface water with salinity and temperature intermediate between them. The chlorophyll-*a* concentration in the mixed water is apparently higher than that in the surface water or the subsurface water. Group C covered quite a large area in the west of our study area. It was influenced by not only the diluted water but also the surface water, showing a preference for high water temperature and chlorophyll-*a* concentration. Larger naked species, *Omegastrombidium jankowskii* and *Strombidium conicum*, were dominant in Group C. Meanwhile, *Mesodinium rubrum*, as a typical autotroph, was abundant in this group. The bottom water in station 2, different from other stations nearby, was characterized by low levels of temperature and chlorophyll-*a*. The species composition here was distinctive. The special nature of station 2 was speculated to be resulted from the diluted water from Vietnam, however, more evidence was needed for further confirmation.

The outer surface water is pushed northward along the western coast of Hainan Island after coming into the gulf [16]. It joins the Qiongzhou Strait West Current and then flows northward along the western coast of Leizhou Peninsula. A high level of nutrients, which may be brought by Qiongzhou Strait West Current from the east coast of Leizhou Peninsula, was detected in the waters west of Leizhou Peninsula. Group A in this area showed a preferrence to high concentration of nutrients and was dominated by loricate species *Tintinnidium primitivum*.

## Conclusion

The ciliate communities in summer in the northern Beibu Gulf, South China Sea showed significant spatial variations of species composition, abundance, biomass and diversity. The variation of ciliate communities was significant at horizontal level, but that was not at vertical level. The spatial pattern of ciliate communities was closely related to certain environmental factors, among which phosphorus, nitrogen and chlorophyll-*a* were the controlling factors in summer in this area. Our results will provide basic data for further studies in ciliate community structure and in aquatic ecology in the Beibu Gulf.

## Supporting Information

Table S1 **List of the ciliate species recorded in multilayer samples from the northern Beibu Gulf.**
(DOC)
