# Supplementary material for: Phosphorus, Nitrogen and Chlorophyll-a Are Significant Factors Controlling Ciliate Communities in Summer in the Northern Beibu Gulf, South China Sea
Source: PLoS One. 2014 Jul 2;9(7):e101121. doi: 10.1371/journal.pone.0101121 (PMC4079230; doi:10.1371/journal.pone.0101121)
Supplement: Table S1 — List of the ciliate species recorded in multilayer samples from the northern Beibu Gulf. (DOC) [file pone.0101121.s001.doc]

**Table S1. List of the ciliate species recorded in multilayer samples from the northern Beibu Gulf.**

| **Species** | **Abundance^1^**  **(ind·L^-1^)** | | | **Biomass^2^**  **(ng C·L^-1^)** | | | **Body size^3^**  **(µm)** | | | **Frequency of occurrence ^4^** |
| --- | --- | --- | --- | --- | --- | --- | --- | --- | --- | --- |
| **Oligotrichida** |  |  |  |  |  |  |  |  |  |  |
| *Apostrombidium pseudokielum* | 4.11 | ± | 2.91 | 2.13 | ± | 1.43 | 20~22.5 | × | 15 | 17.74% |
| *Halteria grandinella* | 45.54 | ± | 11.29 | 4.72 | ± | 1.21 | 10~15 | × | 10~15 | 45.16% |
| *Laboea strobila* | 116.55 | ± | 27.75 | 353.06 | ± | 78.12 | 35~70 | × | 25~47.5 | 59.68% |
| *Leegaardiella ovalis* ^[7]^ | 174.98 | ± | 38.99 | 101.47 | ± | 26.13 | 7.5~25 | × | 10~25 | 61.29% |
| *Leegaardiella* sp. | 5.56 | ± | 3.09 | 24.73 | ± | 13.64 | 40~60 | × | 40~45 | 17.74% |
| *Novistrombidium testaceum* | 0.24 | ± | 0.24 | 7.45 | ± | 7.45 | 55 | × | 75 | 14.52% |
| *Omegastrombidium jankowskii* | 40.77 | ± | 8.40 | 413.14 | ± | 90.33 | 60~120 | × | 30~70 | 48.39% |
| *Parallelostrombidium paralatum* | 0.24 | ± | 0.24 | 0.29 | ± | 0.29 | 30 | × | 20 | 14.52% |
| *Parastrombidium faurei* | 0.24 | ± | 0.24 | 5.85 | ± | 5.85 | 67.5 | × | 60 | 14.52% |
| *Pelagostrobilidium simile* | 0.73 | ± | 0.54 | 0.48 | ± | 0.40 | 17.5~30 | × | 15~30 | 16.13% |
| *Rimostrombidium caudatum* | 101.92 | ± | 19.35 | 26.22 | ± | 5.01 | 17.5~25 | × | 12.5~17.5 | 59.68% |
| *Rimostrombidium kahli* | 0.24 | ± | 0.24 | 0.26 | ± | 0.26 | 35 | × | 25 | 14.52% |
| *Rimostrombidium orientale* | 0.24 | ± | 0.24 | 0.11 | ± | 0.11 | 20 | × | 15 | 14.52% |
| *Spirostrombidium agathae* | 7.38 | ± | 4.08 | 3.74 | ± | 2.32 | 15~22.5 | × | 12.5~17.5 | 19.35% |
| *Spirostrombidium cinctum* | 26.37 | ± | 8.02 | 23.69 | ± | 6.77 | 22.5~27.5 | × | 15~20 | 30.65% |
| *Spirostrombidium schizostomum* | 1.21 | ± | 0.72 | 2.08 | ± | 1.21 | 30~50 | × | 17.5~27.5 | 17.74% |
| *Spirotontonia turbinata* | 27.40 | ± | 7.53 | 24.81 | ± | 6.49 | 27.5~50 | × | 20~25 | 40.32% |
| *Strobilidium neptuni* | 0.97 | ± | 0.58 | 7.86 | ± | 5.67 | 45~50 | × | 30~50 | 17.74% |
| *Strobilidium sphaericum* | 2.48 | ± | 1.17 | 9.05 | ± | 4.86 | 15~40 | × | 25~40 | 20.97% |
| *Strobilidium spiralis* | 40.34 | ± | 8.96 | 99.99 | ± | 20.97 | 25~45 | × | 25 | 53.23% |
| *Strombidinopsis acuminatum* | 23.59 | ± | 6.42 | 28.86 | ± | 7.63 | 25~45 | × | 20~35 | 38.71% |
| *Strombidinopsis cheshiri* | 3.51 | ± | 3.04 | 18.69 | ± | 16.18 | 45~90 | × | 20~40 | 16.13% |
| *Strombidinopsis elegans* | 19.90 | ± | 7.33 | 13.33 | ± | 4.58 | 17.5~25 | × | 15~20 | 27.42% |
| *Strombidinopsis elongata* | 4.35 | ± | 2.50 | 10.09 | ± | 5.61 | 50~67.5 | × | 22.5~37.5 | 19.35% |
| *Strombidium acutum* | 157.68 | ± | 43.36 | 77.74 | ± | 20.12 | 10~25 | × | 10~22.5 | 48.39% |
| *Strombidium apolatum* | 2.90 | ± | 1.42 | 2.38 | ± | 1.23 | 25~27.5 | × | 12.5~15 | 20.97% |
| *Strombidium capitatum* | 70.28 | ± | 10.12 | 105.26 | ± | 25.68 | 20~30 | × | 20~27.5 | 69.35% |
| *Strombidium conicum*^[5]^ | 206.07 | ± | 31.08 | 204.10 | ± | 31.58 | 27.5~55 | × | 15~30 | 83.87% |
| *Strombidium emergens* ^[8]^ | 170.63 | ± | 32.94 | 112.87 | ± | 24.14 | 25~37.5 | × | 15~25 | 56.45% |
| *Strombidium globosaneum* | 83.17 | ± | 13.20 | 16.35 | ± | 2.68 | 12.5~17.5 | × | 10~15 | 67.74% |
| *Strombidium inclinatum* ^[2]^ | 520.40 | ± | 140.74 | 111.68 | ± | 27.87 | 10~22.5 | × | 7.5~20 | 82.26% |
| *Strombidium montagnesi* | 5.02 | ± | 2.04 | 3.63 | ± | 1.60 | 20~22.5 | × | 15~17.5 | 22.58% |
| *Strombidium paracalkinsi* | 62.66 | ± | 16.82 | 210.42 | ± | 140.39 | 15~55 | × | 12.5~45 | 48.39% |
| *Strombidium parastylifer* ^[4]^ | 232.32 | ± | 28.84 | 38.07 | ± | 5.59 | 12.5~25 | × | 10~20 | 87.10% |
| *Strombidium stylifer* | 97.86 | ± | 23.15 | 114.30 | ± | 29.20 | 22.5~45 | × | 17.5~25 | 61.29% |
| *Strombidium sulcatum* | 113.71 | ± | 20.52 | 65.78 | ± | 11.08 | 17.5~25 | × | 12.5~22.5 | 69.35% |
| *Strombidium tintinnodes* | 116.37 | ± | 24.53 | 36.70 | ± | 7.50 | 17.5~25 | × | 12.5~17.5 | 62.90% |
| *Strombidium typicum* | 0.24 | ± | 0.24 | 0.53 | ± | 0.53 | 35 | × | 25 | 14.52% |
| *Strombidium wilberti* | 53.77 | ± | 10.32 | 22.54 | ± | 4.38 | 17.5~25 | × | 15~22.5 | 51.61% |
| *Varistrombidium kielum* | 0.48 | ± | 0.48 | 1.09 | ± | 1.09 | 35~55 | × | 17.5~25 | 14.52% |
| **Scuticociliatida** |  |  |  |  |  |  |  |  |  |  |
| *Eurystoma sinicum* | 0.73 | ± | 0.41 | 0.33 | ± | 0.23 | 30~35 | × | 20 | 14.52% |
| *Miamiensis avidus* | 0.48 | ± | 0.48 | 0.63 | ± | 0.63 | 15~30 | × | 10~17.5 | 17.74% |
| *Paranophrys marina* | 0.24 | ± | 0.24 | 0.07 | ± | 0.07 | 27.5 | × | 10 | 14.52% |
| *Pardlembus digitiformis* | 7.26 | ± | 2.26 | 4.86 | ± | 1.54 | 20~27.5 | × | 15~22.5 | 30.65% |
| *Philasterides armatalis* | 0.24 | ± | 0.24 | 0.04 | ± | 0.04 | 15 | × | 10 | 14.52% |
| *Uronema elegans* | 0.85 | ± | 0.65 | 2.18 | ± | 1.91 | 30~35 | × | 20~39 | 14.52% |
| *Uronemella binucleata* | 0.24 | ± | 0.24 | 0.04 | ± | 0.04 | 15 | × | 10 | 14.52% |
| *Uronemella filificum* | 7.86 | ± | 2.71 | 2.64 | ± | 0.87 | 1.5~22.5 | × | 10~15 | 25.81% |
| **Pleurostomatida** |  |  |  |  |  |  |  |  |  |  |
| *Amphileptus sikorai* | 0.30 | ± | 0.30 | 0.80 | ± | 0.80 | 42.5 | × | 25 | 12.90% |
| **Haptorida** |  |  |  |  |  |  |  |  |  |  |
| *Didinium balbianni* | 2.42 | ± | 1.20 | 8.53 | ± | 3.93 | 35~45 | × | 22.5~35 | 20.97% |
| *Didinium gargantua* | 1.94 | ± | 1.22 | 25.72 | ± | 18.93 | 40~100 | × | 25~75 | 17.74% |
| *Mesodinium pulex* | 22.26 | ± | 11.13 | 4.37 | ± | 2.08 | 10~20 | × | 7.5~15 | 27.42% |
| *Mesodinium rubrum*^[1]^ | 702.58 | ± | 106.08 | 231.27 | ± | 41.36 | 10~32.5 | × | 10~27.5 | 93.55% |
| *Mesodinium velox*^[3]^ | 247.14 | ± | 45.05 | 39.07 | ± | 7.69 | 15~30 | × | 10~17.5 | 85.48% |
| **Euplotida** |  |  |  |  |  |  |  |  |  |  |
| *Euplotes* sp. | 1.69 | ± | 1.30 | 3.82 | ± | 3.00 | 40~50 | × | 22.5~25 | 16.13% |
| *Euplotes vannus* | 0.91 | ± | 0.91 | 1.50 | ± | 1.50 | 40~45 | × | 20 | 12.90% |
| **Sessilida** |  |  |  |  |  |  |  |  |  |  |
| *Vorticella* sp. | 77.24 | ± | 47.54 | 88.41 | ± | 56.19 | 20~25 | × | 20~25 | 20.97% |
| *Zoothamnium* sp. | 18.15 | ± | 18.15 | 9.03 | ± | 9.03 | 25 | × | 20 | 14.52% |
| **Tintinnida** |  |  |  |  |  |  |  |  |  |  |
| *Amphorellopsis acuta* | 39.19 | ± | 8.70 | 175.37 | ± | 39.79 | 90~110 | × | 25~30 | 41.94% |
| *Codonella rapa* | 0.24 | ± | 0.24 | 0.40 | ± | 0.40 | 55 | × | 30 | 14.52% |
| *Codonellopsis morchella* | 0.48 | ± | 0.34 | 4.94 | ± | 3.75 | 100~102.5 | × | 35~37.5 | 16.13% |
| *Cyttarocylis eucecryphalus* | 1.21 | ± | 1.21 | 2.25 | ± | 2.25 | 35 | × | 40 | 14.52% |
| *Dadayiella ganymedes* | 0.79 | ± | 0.57 | 4.02 | ± | 3.00 | 95~110 | × | 30~32.5 | 14.52% |
| *Eutintinnus apertus* | 2.66 | ± | 2.43 | 0.89 | ± | 0.81 | 35~45 | × | 10~15 | 16.13% |
| *Eutintinnus inquilinus* | 1.45 | ± | 1.07 | 2.70 | ± | 1.92 | 75~85 | × | 15~22.5 | 16.13% |
| *Eutintinnus lusus-undae* | 0.48 | ± | 0.34 | 6.48 | ± | 4.60 | 185~195 | × | 35~40 | 16.13% |
| *Favella ehrenbergii* | 0.24 | ± | 0.24 | 13.91 | ± | 13.91 | 160 | × | 85 | 14.52% |
| *Leprotintinnus bottnicus* | 0.73 | ± | 0.73 | 9.72 | ± | 9.72 | 160~185 | × | 40 | 14.52% |
| *Leprotintinnus nordquisti* | 0.73 | ± | 0.54 | 12.68 | ± | 9.75 | 180~240 | × | 35~45 | 16.13% |
| *Leprotintinnus simplex* | 20.63 | ± | 11.09 | 161.60 | ± | 74.14 | 60~305 | × | 35~50 | 22.58% |
| *Metaclylis oviformis* | 15.30 | ± | 5.88 | 31.74 | ± | 12.22 | 50~60 | × | 30~35 | 24.19% |
| *Metacylis corbula* | 1.94 | ± | 1.31 | 5.64 | ± | 3.77 | 40~45 | × | 45~47.5 | 17.74% |
| *Metacylis mereschkowskii* | 0.24 | ± | 0.24 | 0.57 | ± | 0.57 | 35 | × | 45 | 14.52% |
| *Proplectella acuta* | 2.66 | ± | 1.97 | 4.18 | ± | 3.40 | 45~55 | × | 30~35 | 19.35% |
| *Rhabdonella sanyahensis* | 0.97 | ± | 0.76 | 3.82 | ± | 2.94 | 110~130 | × | 45 | 16.13% |
| *Stenosemella nivalis* | 9.92 | ± | 4.12 | 29.95 | ± | 12.08 | 40~50 | × | 35~50 | 24.19% |
| *Stenosemella pacifica* | 1.45 | ± | 0.89 | 1.27 | ± | 0.79 | 30~37.5 | × | 25~32.5 | 17.74% |
| *Stenosemella parvicollis* | 22.68 | ± | 10.02 | 96.91 | ± | 43.52 | 50~55 | × | 40~52.5 | 29.03% |
| *Tintinnidium primitivum*^[6]^ | 330.30 | ± | 103.65 | 524.87 | ± | 167.15 | 40~150 | × | 20~25 | 51.61% |
| *Tintinnopsis aperta* | 0.73 | ± | 0.54 | 1.03 | ± | 0.78 | 65~75 | × | 20 | 16.13% |
| *Tintinnopsis beroidea* | 0.24 | ± | 0.24 | 0.65 | ± | 0.65 | 60 | × | 30 | 14.52% |
| *Tintinnopsis bütschlii* | 1.45 | ± | 0.89 | 2.12 | ± | 1.46 | 50~60 | × | 65~75 | 17.74% |
| *Tintinnopsis gracilis* | 5.81 | ± | 2.68 | 42.09 | ± | 20.05 | 100~145 | × | 30~35 | 19.35% |
| *Tintinnopsis karajacensis* | 1.39 | ± | 0.96 | 4.70 | ± | 2.87 | 57.5~60 | × | 30~35 | 16.13% |
| *Tintinnopsis karajacensis* var. *rotundata* | 2.78 | ± | 1.71 | 5.10 | ± | 3.16 | 45~52.5 | × | 25~30 | 16.13% |
| *Tintinnopsis kofoidi* | 9.92 | ± | 4.02 | 89.67 | ± | 38.09 | 90~165 | × | 25~40 | 25.81% |
| *Tintinnopsis lobiancoi* | 8.23 | ± | 4.32 | 68.63 | ± | 37.01 | 117.5~150 | × | 30~40 | 20.97% |
| *Tintinnopsis lohmanni* | 0.24 | ± | 0.24 | 0.43 | ± | 0.43 | 40 | × | 30 | 14.52% |
| *Tintinnopsis minuta* | 5.44 | ± | 2.82 | 3.26 | ± | 1.57 | 25~40 | × | 20~30 | 20.97% |
| *Tintinnopsis nana* | 9.19 | ± | 3.09 | 6.35 | ± | 2.11 | 25~35 | × | 20 | 27.42% |
| *Tintinnopsis nucula* | 0.73 | ± | 0.54 | 0.92 | ± | 0.67 | 37.5~45 | × | 25 | 16.13% |
| *Tintinnopsis radix* | 2.18 | ± | 1.95 | 48.63 | ± | 42.42 | 145~490 | × | 35~47.5 | 16.13% |
| *Tintinnopsis schotti* | 31.15 | ± | 13.15 | 997.00 | ± | 415.73 | 90~115 | × | 70~85 | 30.65% |
| *Tintinnopsis sufflata* | 0.24 | ± | 0.24 | 0.53 | ± | 0.53 | 70 | × | 25 | 14.52% |
| *Tintinnopsis tenuis* | 6.05 | ± | 2.83 | 10.23 | ± | 4.23 | 55~65 | × | 22.5~25 | 24.19% |
| *Tintinnopsis tocantinensis* | 25.40 | ± | 8.17 | 62.48 | ± | 21.31 | 85~120 | × | 20~25 | 32.26% |
| *Tintinnopsis tubulosa* | 0.48 | ± | 0.48 | 1.81 | ± | 1.81 | 82.5~85 | × | 30 | 14.52% |
| *Tintinnopsis tubulosoides* | 1.45 | ± | 1.02 | 10.42 | ± | 7.55 | 80~85 | × | 35~37.5 | 16.13% |
| *Tintinnopsis urnula* | 3.69 | ± | 2.23 | 8.34 | ± | 4.79 | 40~60 | × | 30~37.5 | 17.74% |
| *Undella ostenfeldi* | 0.48 | ± | 0.48 | 0.25 | ± | 0.25 | 45 | × | 25~27.5 | 14.52% |
| *Undella* sp. | 0.24 | ± | 0.24 | 1.25 | ± | 1.25 | 85 | × | 35 | 14.52% |

^1^average abundance ± SE (ind·L^-1^); ^2^average biomass ± SE (ng C·L^-1^); ^3^body size (µm) (length × width); ^4^frequency of occurrence (%) = number of occurred samples/total number of samples (62); numbers in the superscript square brackets = ranks of 8 dominant ciliates (*Y* >0.02).
